# Application of Pin-On-Disc Techniques for the Study of Tribological Interferences in the Dry Machining of A92024-T3 (Al–Cu) Alloys

**DOI:** 10.3390/ma11071236

**Published:** 2018-07-18

**Authors:** Jorge Salguero, Juan Manuel Vazquez-Martinez, Irene Del Sol, Moises Batista

**Affiliations:** Mechanical Engineering & Industrial Design Department Faculty of Engineering, University of Cadiz, Av. Universidad de Cadiz 10, E-11519 Puerto Real-Cadiz, Spain; juanmanuel.vazquez@uca.es (J.M.V.-M.); irene.delsol@uca.es (I.D.S.); moises.batista@uca.es (M.B.)

**Keywords:** UNS A92024, WC–Co, wear mechanism, tribology, adhesion

## Abstract

One of the main criteria for the establishment of the performance of a forming process by material removal is based on cutting tool wear. Wear is usually caused by different mechanisms, however, only one is usually considered as predominant or the controller of the process. This experimental research is focused on the application of Pin-on-Disc wear tests, in which the tribological interference between UNS A92024-T3 Aluminum–Copper alloy and tungsten carbide (WC–Co) has been studied. The main objective of this study is focused on the determination of the predominant wear mechanisms involved in the process, as well as the characterization of the sliding and friction effects by using SEM and Energy Dispersion Spectroscopy (EDS) techniques, as applied to WC–Co (cutting tool material)/Al (workpiece material) which are widely used in the aerospace industry. Performed analysis prove the appearance of abrasive wear mechanisms prior to adhesion. This fact promotes adhesion mechanisms in several stages because of the surface quality deterioration, presenting different alloy composition in the form of a Built-Up Layer (BUL)/Built-Up Edge (BUE).

## 1. Introduction

Light alloys, mainly aluminum and titanium, are commonly used in different manufacturing fields especially because of their high performance—weight rate, their excellent physical-chemical properties and their advantageous economic cost. In this context, the aluminum market has greatly benefited thanks to its wide application in the aerospace industry, among others.

Continuous growth in the use of these alloys, and constant requirements to improve the performance of their manufacturing processes follow an important tendency to gradually increase the development of different research lines. The aim of these is to find the optimum conditions for the forming procedures of aluminum alloys.

Material removal, or machining, is among the main applications of aluminum alloy forming processes for aerospace applications. However, in the last decades machining processes have been characterized by a reorientation towards less aggressive environmental processes by means of the minimization or elimination of the use of cutting fluids in the cutting process. Under these conditions, cutting tools show an intensification of their wear mechanisms, causing deviations on the initial design specifications in the machined part [1,2]. For this reason, preserving the initial geometry of the cutting tool as long as possible is essential to ensure final tolerances [3]. In this context, a preliminary study of the tribological interference between the material being machined and tools is critical to maintain a precise control of process parameters, obtaining higher performance ranges.

**Figure 1:**
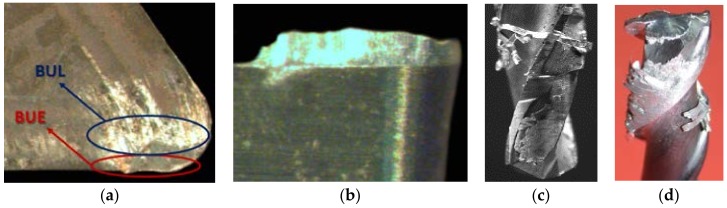
Cutting tools affected by adhesive wear mechanism in the machining of Al alloys: (**a**) turning insert tool; (**b**) detail of the adhered material thickness; (**c**) drilling tool; (**d**) milling tool for radial operations.

In general terms, when aluminum alloys are machined by chip removal processes, the tool wear process is mainly characterized by the appearance of secondary or indirect adhesion mechanisms [4]. This wear mechanism is specifically based on the incorporation of the machined material over two well-localized areas of the cutting tool: at the edge, giving rise to the Built-Up Edge (BUE); and at the rake face, giving rise to a Built-Up Layer (BUL). Both types of material incorporation may modify the initial cutting geometry, affecting the surface quality of the machined parts [5], as is mentioned previously, and can be seen in the macrographs of Figure 1. In addition, the mechanical instability of the involved effects tends to cause a friction process promoted by the chip, resulting in the lost of particles of the tool surface, which constitutes the main wear effect [6].

Tool wear mechanisms can be studied under lab conditions using Pin-on-Disk or Pin-on-Flat tests [7,8,9,10] reducing the material cost and improving the environmental sustainability of the process. However, only a few studies have been found which investigate the specific tribological pairing of aluminum and tungsten carbide [11]. For this reason, the present work is focused on the study of the wear mechanisms involved in the tribological friction and sliding conditions of the WC–Co (tool material) and UNS A92024-T3 aluminum alloy. Pin-on-Disc test techniques were performed and wear effects were analyzed by volume variation and SEM/Energy Dispersion Spectroscopy (EDS) microscopy in order to obtain a deeper understanding for the detected wear phenomena.

## 2. Materials and Methods

**Figure 2:**
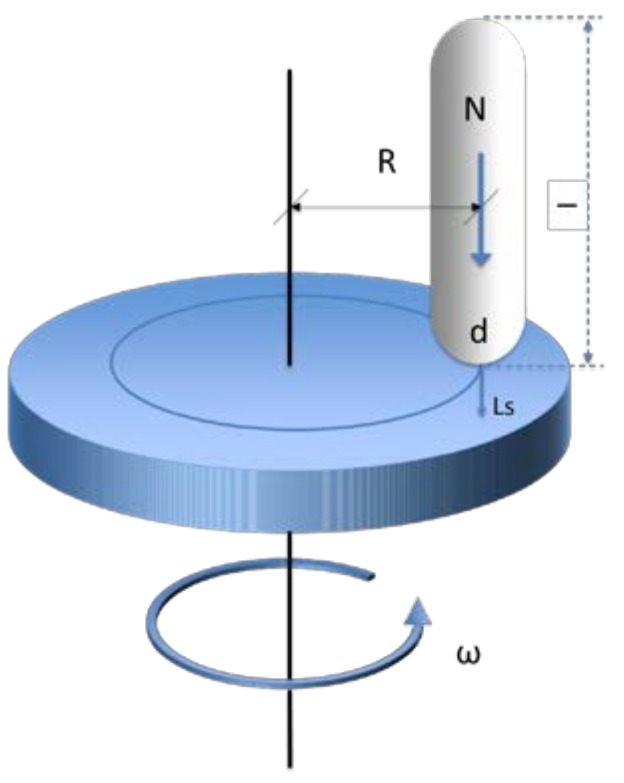
Pin-on-Disc tribometer motion diagram.

Pin-on-Disc tribological tests (PoD) were carried out under dry conditions, using a MT/60/NI Microtest Tribometer (Microtest S.A., Madrid, Spain) (motion diagram in Figure 2). The load (N) and linear speed (Ls), track radius (R) and turning speed (ω) were constant, while sliding distance varied, as is listed in Table 1. During the test development, the dynamometric response values, environmental conditions, and temperatures were measured.

Firstly, Al–Cu UNS A92024-T3 (Ra < 0.4 μm) samples of 90 × 90 mm^2^ and thicknesses between 1.6 and 2.0 mm were selected as discs. Their composition is shown in Table 2.

Then, (WC-6%Co) carbide metal bars with 30 mm length (l) and 4 mm diameter (d) hemispherical ends were used to simulate tool displacement (pins). The average and maximum Hertz contact pressure for the tribological pair were calculated as 0.91 and 1.37 GPa, respectively.

Wear evaluation was carried out following the guidelines of the ASTM G99-04 standard, expressing the friction effects in terms of material volume loss (mm^3^) as a function of the sliding length. All samples were carefully cleaned using petroleum ether and alcohol (50%). The weight of the aluminum probes were evaluated by a precision scale (Ohaus Pioneer PA214, Parsippany, NJ, USA) before (P0) and after (PF) the development of the tribotests. The precision scale used in the weight evaluation of the samples have a 0.0001 g resolution. This scale resolution is the recommended by the ASTM G99 for the evaluation of results in Pin-on-Disc tribological tests.

In addition, visual inspection was carried out by optical microscopy techniques, using a stereoscopic microscopy device (Nikon SMZ-800, Tokyo, Japan) with the aim of analyzing the effects and consequences of the wear mechanisms involved in the process. The wear track was also measured by a profilometer Taylor Hobson Form Talysurf Series 2 (Leicester, UK).

Specific areas of the carbide pins and aluminum discs were established to perform deeper evaluation with a Scanning Electron Microscopy (SEM) and Energy Dispersion Spectroscopy (EDS) microcompositional characterization, by using a FEI Quanta 200 (ThermoFisher Scientific, Hillsboro, OR, USA) with EDAX Phoenix.

## 3. Results and Discussion

The friction coefficient behavior and wear mechanisms involved in the process were studied on the contact surface of the carbide pins. The tribological wear effects were evaluated through volume loss of aluminum discs as a function of sliding length. Additionally, a study on the tribological wear behavior between empirical and theoretical models was carried out taking the Archard coefficient as control parameter.

### 3.1. Friction Coefficient Evaluation

**Figure 3:**
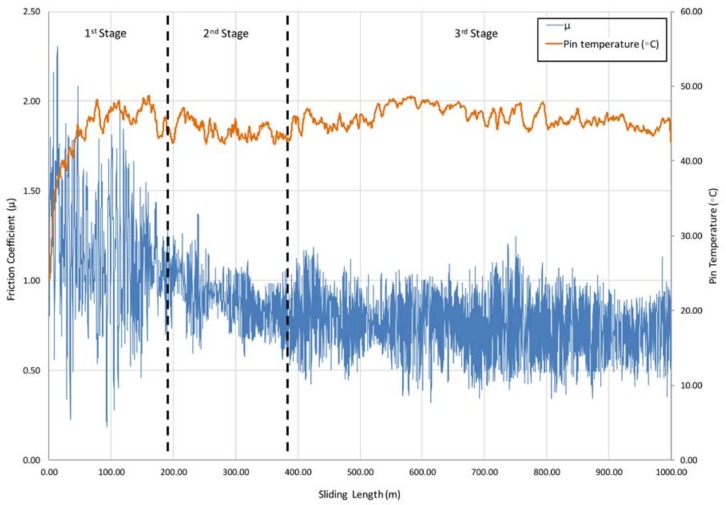
Friction coefficient and pin temperature as a function of sliding length for 1000 m.

The friction coefficient (μ) of the specimens shows specific behavior for different sliding lengths. These phenomena may be mainly caused by the wear mechanisms involved in each stage of the friction process. In this way, through analyzing the obtained values for the friction coefficient, three stages were detected, with different behavior during the sliding course, Figure 3.

**Figure 4:**
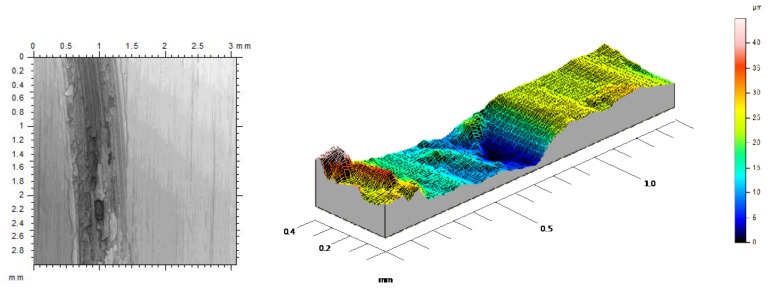
First stage sliding track with abrasive wear effects.

The first stage is observed up to 200 m of sliding length, where the “stick-slip” phenomena takes place. This effect is developed in the dynamic contact between both surfaces, resulting in unstable movements along the sliding track. In the first friction instant, a significant increase in the contact force and μ were caused by the detachment of initial asperities which come from the surface material of the tribological pair. These asperities may cause specific roughness values, leading to a smaller contact surface between the pin and the disc, resulting in higher contact pressures. The action of high contact pressures on rough surfaces tend to remove soft asperities, favoring the detachment of wear debris on the sliding track, and giving place to high amplitude in the friction coefficient values and the appearance of the effects of abrasive wear mechanisms. In this aspect, an important increase of amplitude values implies a growth in the instability during the initial length of the process, especially because of the lack of uniformity of the circular trace, Figure 4.

The increase in μ values is mainly caused by the movement of the pin over wear debris from initial aluminum asperities, involving a quick growth of the temperature in the contact area. A combination of friction and temperature conditions also favor adhesive phenomena from the aluminum particles to carbide surface.

**Figure 5:**
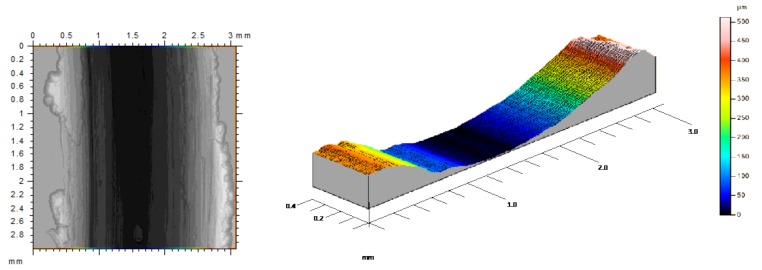
Second stage sliding track with abrasive wear effects.

The next stage starts with a decrease in μ values and a stabilization of the pin temperature. An important reduction in the oscillations amplitude of μ was also detected. This behavior is mainly due to a growth of the adhesion layer of aluminum alloy formed from the slip track over the pin in stratified sections. Under these conditions, adhesion of wear debris over the slip track and pin surface induces the modification towards softer topographies between contact elements, resulting in a sliding process where surfaces of the same material contact each other, Figure 5.

**Figure 6:**
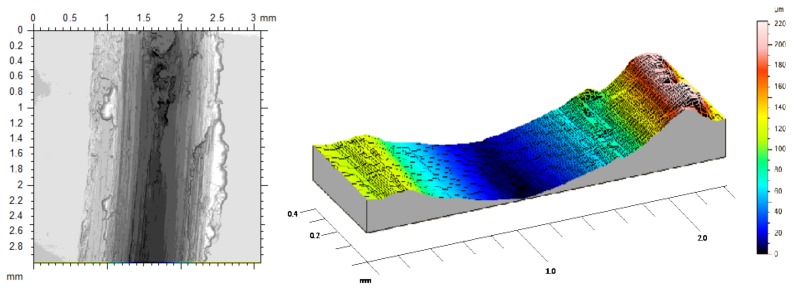
Third stage sliding track, with an adhered debris layer over the sliding way, and deposited material in the outer edges of the track.

In the last stage, the detachment is produced by aluminum adhered debris from the pin surface, as a result of reaching an excessive critical volume. Under these conditions, the adhered layer becomes unstable and may be removed as a consequence of the friction forces on the contact area. In this way, the carbide surface is subjected to wear from the adhered aluminum layer, promoting the lost of particles from the hemispheric surface. Because of this effect, wear debris of aluminum and carbide (harder than the disc) are deposited again over the sliding track. The material debris on the wear trace results in an increase of the μ values and the temperature of the pin, Figure 6. During this stage, the described effect is repeated as a continuous cyclic and dynamic behavior of adhesion wear mechanisms, as is described in previous works [12].

**Figure 7:**
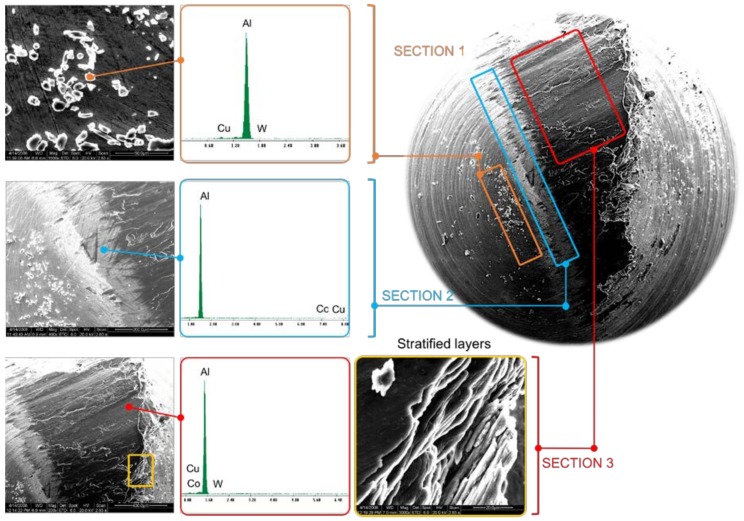
SEM (100×) micrography of the Pin for test 500 m sliding distance. Detailed areas for the adhesion and punctual Energy Dispersion Spectroscopy (EDS) of the different wear phenomena.

When the carbide pin’s surface is analyzed, the existence of three different sections subjected to specific wear mechanisms are observed, Figure 7. In this aspect, similar wear effects are noticed from the morphological adhesion behavior over all of the tested specimens.

Section 1 is especially characterized by the existence of Al–Cu alloy worn particles. These particles have been mechanically adhered to the hemispherical surface, giving place to abrasive wear phenomena in the first instants of the tribological tests.

Section 2 is formed by the primary layer developed in the first instants of the tribological test. EDS analysis shows that the composition of this layer is close to pure Al. According to previous research [13], this adhesive mechanism is mainly associated with a thermomechanical effect.

Section 3 is composed of the secondary layer, specifically described by a stratification of wear debris which is adhered over the primary layer through mechanical effects due to thermomechanical phenomena. Cu composition percentages near to 2.65% were observed.

### 3.2. Wear Effects Evaluation

The material volume loss was selected as the control parameter in order to study the sliding wear effects on the aluminum specimens. In this way, weight variations caused by the Pin-on-Disc test were measured on test probes with different sliding length configurations. The material volume loss was obtained by using the aluminum alloy density:

(1) $$∆V=\;\frac{∆w}{\rho_{A}}\;=\;\frac{w_{F}-w_{0}}{\rho_{A}}\;$$

where $w_{F}$ is the weight of the tested samples, $w_{0}$ is the weight of the samples before the sliding test, and $\rho_{A}$ is the UNSA92024 aluminum alloy density.

In order to simplify analysis considerations and following the indications of the ASTM G99 Standard [6], the wear volume loss was considered negligible for the harder material (WC–Co). However, adhesive wear phenomena from the aluminum discs to carbide hemispherical pins have been evaluated 

**Figure 8:**
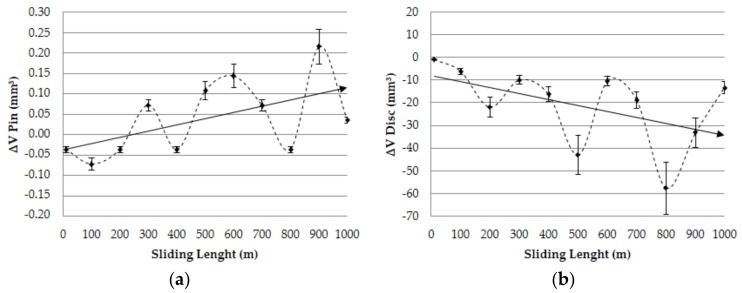
Volume variation vs. sliding length: (**a**) pin; (**b**) disc.

Figure 8 shows the analysis of the volume variation for different samples as a function of sliding length ($L_{d})$.

As was expected, a relevant increase in the values of volume lost for the aluminum discs was detected as a function of sliding length, fitting to a linear behavior and confirming previous research findings [7]. Regarding the pins, an estimation of the main wear mechanism was proposed. On the basis of the results indicated in the Figure 3, an important growth tendency was detected for the adhered material volume on the pins contact surface as a function of the sliding length. This fact may corroborate the raised hypothesis about the importance of secondary adhesion mechanisms in the analyzed tribological pair, detecting a progressive increase of the wear effects regarding the interaction time.

**Figure 9:**
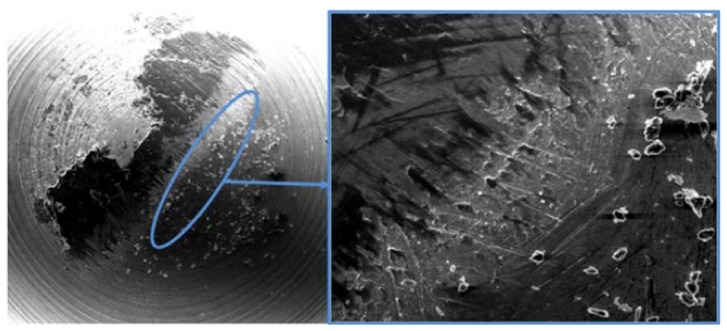
SEM (90×) micrography of the pin for test condition 900 m sliding distance. Detail of the abrasion produced by particles drag (600×).

The appearance in the first instants of several negative values in the variation of volume of the pins should be noticed; this indicates the existence of a slight abrasive wear process prior to the adhesive phenomena. In this respect, SEM confirmed this behavior (Figure 9).

This wear effect was observed in the first instant of sliding tests, as has been commented previously, in which the stick-slip phenomena takes place. The abrasive wear effect finishes when the surface tension of the material breaks, stabilizing the forces involved in the process and allowing the appearance of the first stage of adhesive mechanisms [14]. Furthermore, from the registered data in the tests, the Archard wear ratio [15] was determined, using the following expression:

(2) $$\;K_{s}\;=\;a\cdot L_{d}^{-1}\;$$

where $K_{s}$ is the Archard coefficient, $L_{d}$ is the sliding distance and:

(3) $$\;a\;=\;\frac{∆V\cdot H}{10^{3}\cdot N}\;$$

where ∆V is the volume variation, and H is the softer material hardness [16].

The coefficient exposed in Equation (2) allowed us to carry out the marginal analysis of Archard ratio as a function of the sliding length, taking special care of the fact that the proportionality coefficient is not a constant value, where direct [∆V, $L_{d}$] and indirect variables [H, N] are involved. In fact, the initial hardness of the material may vary as the sliding distance increases, mainly because of a superficial softening effect on the material, favored by the temperature increase in the contact area.

These considerations can differentiate the theoretical model from the real conditions. For this reason, marginal studies can be carried out by the approximation of the experimental results to an empirical model. In this way, a potential model has been selected, following the research lines with a specific interest in material removal [17,18,19,20].

(4) $$\;K_{s}^{'}\;=\;a^{\prime }\times L_{d}^{b}\;$$

This equation can be linearity expressed by logarithmic expression:

(5) $$\;logK_{s}^{'}\;=\;log\;a^{'}+b\times log\;L_{d}↔y\;=\;m\times x+n\;$$

**Figure 10:**
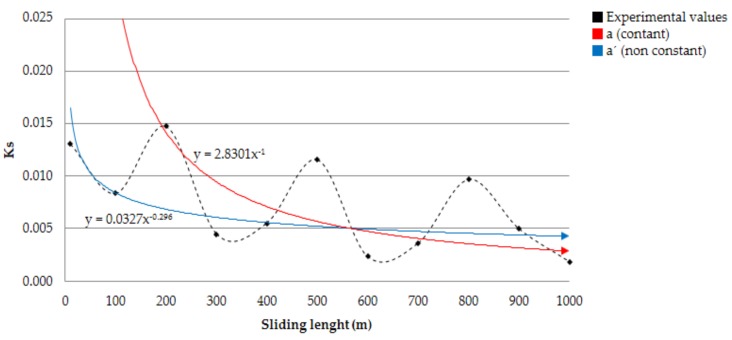
Archard wear coefficient vs. sliding distance.

The potential model is shown in Figure 10 as a function of sliding distance for the different *K_s_* coefficients obtained from empirical and theoretical models.

Comparing the Archard theoretical model (*Ks*) and empirical model (*Ks’*), a relevant difference can be observed in the exponent which govern the sliding length. This disparity may be justified by the existence of specific wear mechanisms that are not taken into account in the theoretical model.

Material hardness (*H*), normal load (*N*), and wear volume (Δ*V*) are considered constant in this model. However, these components are direct or indirect variables of the process, making a  coefficient not constant.

**Figure 11:**
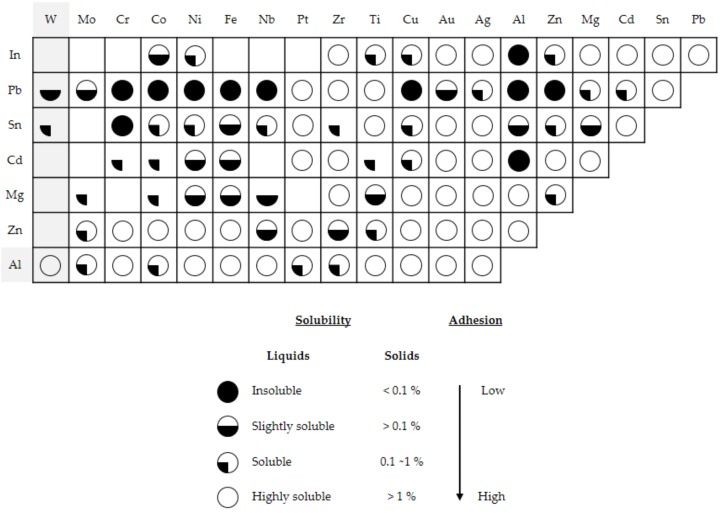
Partial reproduction of the Rabinowicz’s table (adapted from [22]).

In this way, the *Ks* values tendency obtained are located in the Archard range for compatible and/or similar materials subjected to adhesive phenomena [21]. The first consideration may be justified by the friction behavior between the aluminum disc and adhered particles from the alloy to the carbide pins surface. The second one may be justified by the compatibility between Wolfram (W) with Aluminum (Al). This compatibility can be evaluated by the Rabinowicz relation [22], showing higher solubility values (>1%) and a high tendency towards adhesive mechanisms, Figure 11.

## 4. Conclusions

Wear mechanisms are the main responsible factors of cutting tool wear, being present on a wide temperature range. The main mechanism for the tribological pair Al–Cu and WC/Co is secondary adhesion, where the part material is removed and added to the cutting tool surface in the first step. After that, it brings with it the cutting tool’s own particles, increasing the wear effects.

This work studied the tribological interference, simplifying to lab conditions (Pin-on-Disk) of a machining process. This allowed us to isolate the wear due to continuous friction between the contact pair, making it easy to characterize and to verify wear behavior.

The obtained results show an initial abrasion mechanism in the WC/Co pin, which is followed by the secondary adhesion of the aluminum alloy.

This adhesion takes place in two different stages. Firstly, thermomechanical effects (pre-fusion/adhesion) lead to the generation of a thin layer of pure aluminum, which comes from the aluminum matrix. After that, other layers with a similar composition to the Al–Cu alloy are adhered in a stratified way over the first one. This adhesion is due to mechanical effects. With the PoD tests it has been verified that, for pressures close to the ones achieved in finishing machining, the Built-Up Edge and Built-Up Layer effect can be studied for UNS A92024 alloy and WC–Co tribology pair. They are the main wear mechanisms for this pair against abrasion or erosion.

Furthermore, the classical model for the evaluation of Archard wear coefficient do not provide solid results, not taking into account variation of the process as it happens with the superficial hardness of the disc or the orientation of the pin.

## Figures and Tables

**Table 1:** Test Conditions.

Load (N)	Sliding Speed (m/s)	Sliding Distance (m)										
10	1.0	10	100	200	300	400	500	600	700	800	900	1000

**Table 2:** Composition of aluminum–copper alloy (Weight %).

Cu	Mg	Mn	Si	Fe	Zn	Ti	Cr	Al
4.00	1.50	0.60	0.50	0.50	0.25	0.15	0.10	rest
